# Endoscopic litectomy: optimizing the management of sialolithiasis

**DOI:** 10.4317/medoral.26992

**Published:** 2025-03-23

**Authors:** Fernando Almeida-Parra, Álvaro Ranz-Colio, Ángela Bueno-de-Vicente, Cristina Cárdenas-Serres, Patricia de Leyva-Moreno, Julio Acero-Sanz

**Affiliations:** 1 Department of Oral and Maxillofacial Surgery, Ramón y Cajal University Hospital, Madrid, Spain; 2 University of Alcalá, Spain

## Abstract

**Background:**

Sialolithiasis of the salivary glandular complex appears with high frequency in the major salivary glands. The most affected salivary gland is the submandibular gland, followed by the parotid and sublingual glands. The treatment of this disease by endoscopic litectomy has reduced the need of adenectomy.

**Material and Methods:**

We reviewed retrospectively a series of 77 patients who had salivary gland stones between January 2020 and January 2024. Inclusion criteria for endoscopic treatment was lithiasis smaller than 8 mm. Follow-up was performed at a week, one month and three months after the surgery by clinical examination with a mean follow-up of 17 months and CT in selected cases.

**Results:**

Treatment was successful in 69 cases, while in 8 patients the treatment failed, with a successful extraction of the stone in 89.61% of patients. A total of 74 stones were removed from 69 patients. The mean stone size was 6.68 mm (range 3 to 8 mm), located mainly in hilum (*n*=61, 75.60%), median duct (*n*=14, 17.07%) and retrocaruncular (*n*=6, 7.31%). Adenectomy, due to failure of the procedure, was performed in 10 patients, 7 in patients due to lack of recovery of the sialolith and in 3 patients due to postoperative stenosis after removal of the sialolith. Complications involved 6 patients with the presence of stenosis after the lithectomy procedure, in 3 patients it was resolved with endoscopic dilation and in the other 3 an adenectomy was necessary.

**Conclusions:**

Minimally invasive intraoral surgery has high success rate, contributes to reduce the need for glandular radical surgery with a low rate of severe complications.

** Key words:**Sialolithiasis, adenectomy, sialoendoscopy, salivary gland.

## Introduction

Sialolithiasis is classically described as the main cause of salivary glandular obstructive pathology, showing an overall prevalence in postmortem studies of around 1.2% and an incidence of 2.9 to 5.5 cases/100,000 people among the general population, being considered the obstructive cause in 66% of cases according to some authors (1), although recent studies seem to reveal a lower percentage, close to 32% (2).

According to the literature, the incidence of lithiasis is higher between 30 and 60 years, without predominance by sex, being infrequent in children. In the published series, the presence of stones in pediatric age is less than 5% (3).

The presence of lithiasis is more frequent in the submaxillary gland, less frequently in the parotid gland and very rarely in sublingual and minor salivary glands (4). The traditional approach to salivary stones has been open surgical litectomy or in some cases sialoadenectomy. It is estimated that this technique presents a 3% risk of permanent injury to the facial nerve and the possibility of an unaesthetic scar (5).

Sialoendoscopy was introduced from the idea of being able to treat the disease of the salivary ducts from the inside. Small stones, considered up to 8 millimeters in our study, are susceptible to recovery by endoscopic basket extraction. This study presents our 4-year retrospective experience with 77 patients with salivary stones treated using this minimally invasive approach.

## Material and Methods

We retrospectively reviewed the records of all patients with lithiasis in major salivary glands treated by sialoendoscopy between January 2020 and January 2024. The evaluation of the patient was performed by clinical examination that showed little output of saliva to glandular expression, orthopantomography and evidence of the presence of lithiasis in CT scan. The CT made it possible to provide information not only about the sialoliths (size, number and position) but also about the situation of the gland itself. The treatment protocol adopted was to carry out the treatment by minimally invasive technique in cases of sialoliths minor than 8 mm and extraglandular location. Patients with stones larger than 8 mm and those with intraglandular lithiasis were excluded for sialoendoscopy treatment.

In all cases, specific instruments were used allowing the access point to the duct to be dilated and a catheter to be kept in the duct lumen at the same time. Thus, the entrance and exit of the duct is carried out through the solid structure of the catheter. The system used was Kolenda (Cook Medical), which allows to maintain a sTable entry port through which you work without difficulty. It is especially useful in the case of complicated papillae and in the use of lasers, since it requires constant washings and inputs and outputs. The introduction of the access port is preceded by a dilation with the set of dilators.

The clinical variables analyzed were: sex, age, type of gland affected, stone size, number of stones, location of stones (hilar, middle duct, ostium) in terms of their distribution and their possible influence on the extraction of the stone, and the appearance of stenosis as a late complication. We also registered whether or not endoscopic stone extraction was related to glandular preservation. (Table 1) All patients in this series were treated with general anaesthesia. All patients were followed up one week, one month and two months after the surgery.

-Statistical analysis

Statistical analysis was performed on Excel 2013 (Office Professional 2013, Microsoft) and data were analyzed with Python 3.10. Association between extraction of the stone and clinicopathological factors was performed by multivariate analysis using logistic regression analysis. The significant level chosen for all test was *p* < 0.05.

## Results

Between January 2020 and January 2024, a total of 77 subsidiary endoscopic litectomy patients were treated, 37 men and 40 women with a mean age of 44.70 years (median 46 years) (Table 1). In 69 patients (89.61%), extraction of the stones was successfully performed with 74 glandular stones removed by endoscopic approach. 6.15% of patients had more than one sialolith, so in 65 patients one sialolith was extracted, in 3 patients 2 sialoliths were extracted and in one patient 3 were extracted. In the remaining 8 patients (10.39%), the sialoliths could not be extracted by sialoendoscopy. Therefore, our overall success rate for endoscopic lithectomy was 89.61%. In all the patients treated, the visualization of the stone was achieved, trying in all cases the extraction by basket. Of the 8 remaining cases, in 7 sialoadenectomy was performed in a second time and in 1 case the patient declined treatment due to clinical improvement despite evidence of the persistence of the stone.

The mean size of the stones removed was 6.68 mm, and in 25.9% (*n*=20) of patients the stones were greater than 7 mm. The anatomical position of the stones was divided into three main ductal locations, the glandular hilum, the median duct and ostium. In our series, the stones were located in the glandular hilum in 75.60% of cases, in the middle duct in 17.07% of cases and in the ostium in 7.31% of cases. 72 patients (93.5%) were discharged the same day of surgery and the remaining 5, one day after the procedure.

Technique failure was evident in 8 patients, in whom, despite locating and visualizing the stone, it could not be extracted with the basket. Postoperative stenosis complication was present in 6 patients, which was subsequently treated by endoscopic dilation, which resolved the problem in half of the cases (3 cases), requiring the other half adenectomy due to persistence of the clinic (3 cases).

**Figure 1:**
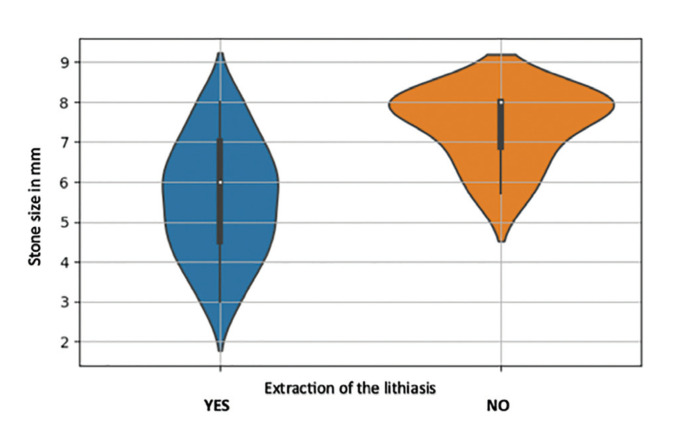
The figure showing that the larger is the size of the stone, the lower is the probability of extraction.

Multivariant analysis including type of gland, sex, age, size of the stone, number of stones and their location was performed showing that both the type of gland and the size of the stone influence the result of extraction of the stone with statistical significance (*p-value* less than 0.05). Specifically, if the stone is located in the submaxillary gland it is approximately 30 times more likely to be removed than if it is located in the parotid. In the case of the size of the stone, the larger the size, the lower the probability of extraction. In our specific case, for every increase of one millimeter in the size of the stone there was 84% less probability of extraction. (Fig. 1) On the other hand, the rest of the clinical variables studied do not seem to be related to the result of stone extraction.

Regarding the onset of stenosis as a complication, the size of the stone has a positive relationship (the larger the size, the greater the probability of stenosis) although it is not statistically significant (*p* = 0.0611)

Related to glandular preservation, stone extraction has a positive relationship with preservation, being statistically significant. It is about 200 times more likely to be preserved if the stone has been extracted.

## Discussion

The annual incidence of salivary lithiasis is estimated between 2,9 to 5,5 cases/100000. Prevalence studies estimate 1.2% postmortem, being considered the obstructive cause in 66% of cases according to some authors (1), although recent studies seem to reveal a lower percentage, close to 32% (2). Lithiasis can occur at any age but the peak of maximum frequency is between the fourth and sixth decade of life, with an average age of 47.7 years (6). These data coincide with those found in our series, whose mean age of patients was 44.70 years with a median of 46 years.

The presence of lithiasis is more frequent in the submaxillary gland (80.4-82%), less frequently in the parotid gland (18-19.6%) and very rarely in minor sublingual and salivary glands (2%) (7). Among the pathophysiological causes that justify the submaxillary predominance, the anatomical ones stand out since the Wharton duct is of greater caliber, with a path with marked curvatures (especially the hilar or mylohyoid), in addition to a flow against gravity and a more viscous saliva (8). The data found in our series shows more prevalence in the submaxillary gland than what has been published in the literature, with 87% of cases of localization in the submaxillary gland and 13% of cases in the parotid gland.

According to publications based on operated patients, lithiasis of parotid origin significantly presents a longer time of evolution of symptoms (median 53.8 months) compared to submaxillary (median 37.9 months). This is due to the more conservative management of parotid localized lithiasis due to the risk of facial nerve injury. Derived from the above fact, the age of the operated patients is significantly higher in parotid stones (mean of 53.7 years) than the submaxillary ones (mean of 44.1 years) (6). That is not present in our series, since the mean age of patients operated on with mandibular lithiasis was 44.85 years, while the mean age of patients with parotid lithiasis was 43.7. This could be due to the small number of cases we present of patients treated for parotid lithiasis.

Lithiasis can be found in the main duct, glandular hilum, or intraglandular ducts. In recent series, the predominant location of sialoliths in the salivary tract was the main duct (74%), followed by the hilar region (22%) (9). In detail, depending on the gland affected, most of the stones that affect the parotid are ductal. However, in cases of the submaxillary gland there is more controversy in the predominant location of the lithiasis. Thus, some series locate the most frequent point of origin of submaxillary lithiasis in the hilum, compared to more recent ones, which locate it in the main duct (10). This was what happened in our series, which being predominantly in the submaxillary gland, we found 75.60% of the stones in the hilum, with the remaining 24.40% being in the main duct.

The conventional treatment of salivary glandular inflammatory pathology of lithiasic origin has been litectomy by incision in the drainage ostium of the salivary ducts when the stone was in an accessible location or by adenectomy when the stone was located in the middle portion of the duct or in the glandular hilum. The risk of papillotomy is stenosis at the level of the ostium. In addition, adenectomy requires hospitalization, produces a scar in the neck or in the parotid region and its technique carries a risk of injury to nerve branches of the facial. This risk increases in parotid surgery when the patient has had previous inflammatory episodes since residual fibrosis increases the surgical difficulty and with it the operative risk.

The exploration of the salivary tree by direct vision was first performed in 1988 (Katz) (11), thanks using ultrathin flexible endoscopes. It is a technique that allows the diagnosis and conservative therapeutic management of obstructive salivary pathology. In this way, it is a minimally invasive technique, reducing iatrogenesis, resulting in significantly lower morbidity for patients. Since it acts on the main ducts, in most cases, it allows the extraction of sialoliths through an intraductal approach without the need to perform the traditional procedures of papillotomy or adenectomy mentioned above. The benefits of this technique are evident, in terms of morbidity and associated quality of life. However, the cornerstone of sialoendoscopy is the learning curve. It is not entirely clear what the number of procedures to be performed to overcome the first part of this curve is, but it seems to point to 30-50 cases, a lower number than that of other endoscopic techniques (12).

The success rate for stone removal in this consecutive case series was 89.61% of patients. These data are close to those obtained in series larger than ours that describe success rates of between 90-95%. In our serie, we found the stone in 100% of the cases, unlike what was found in other series, which report not being able to locate the sialolith in 2.3-3% of cases (11).

Endoscopic removal of sialoliths proved to be a very safe procedure with few complications. No intraoperative complications such as inability to locate the stone, bleeding requiring interrupt of the procedure or false routes, were present in our study. There was no evidence of postoperative infection in any of the patients. The main postoperative complication was the presence of postoperative stenosis in 6 patients (7.79%), which coincides with what has been published in the literature. However, unlike other authors who presented stenosis in cases treated with lithiasis in the hilum, in our series, of the 6 cases, only 2 presented lithiasis in the hilum.

Minimally invasive gland surgery has reduced the need for a gland adenectomy, with only 10 cases needing the gland removal in the current series. This data is similar with the published in the literature, which states that endoscopic lithectomy procedures reduce the need for adenectomy and/or papillotomy by 90% (13).

The average length of hospital stay during the 4-year audit period improved from 1.6 days in the first third of cases to 0.7 days in the last third. This is explained by the fact that most patients who underwent adenectomy were treated in the first third of the study period while most patients treated in the last period resolved favorably with endoscopic lithectomy.

## Conclusions

Sialoendoscopy is a minimally invasive technique that allows the diagnosis and conservative therapeutic management of obstructive salivary pathology of lithiasic origin. In this way, it acts mainly on the ductal tree reducing iatrogenesis and resulting in significantly lower morbidity for patients. In general, minimally invasive management of sialoliths is a practical and successful treatment modality for stones up to 8 mm in diameter located in the salivary ducts. In most cases, glandular removal is avoided, reducing morbidity and associated hospital costs.

## Figures and Tables

**Table 1:** 77 patients treated with clinical variables studied: affected salivary gland, sex, age, stone size in mm, number of stones, location of the stones, whether the stone was removed and whether the gland was preserved after the intervention and/or the appearance of ductal stenosis.

Nº	Gland	Sex	Age	Size (mm)	Number	Localization	Extraction	Gland Preservation	Stenosis
1	SUBMAX	Male	19	4	1	Retrocaruncular	Yes	Yes	No
2	SUBMAX	Female	55	5	1	Hilum	Yes	Yes	No
3	SUBMAX	Male	50	6	1	Hilum	Yes	Yes	No
4	SUBMAX	Male	62	3	1	Hilum	Yes	Yes	No
5	SUBMAX	Male	33	4	1	Median duct	Yes	Yes	No
6	SUBMAX	Male	58	4	1	Median duct	Yes	Yes	No
7	SUBMAX	Female	70	5	1	Hilum	Yes	Yes	No
8	SUBMAX	Female	54	6	1	Hilum	Yes	Yes	No
9	SUBMAX	Female	60	8	1	Hilum	No	No	No
10	SUBMAX	Male	40	7-ene	2	Retrocaruncular	Yes	No	Yes
11	SUBMAX	Female	72	5	1	Hilum	Yes	Yes	No
12	SUBMAX	Female	46	4	1	Hilum	Yes	Yes	No
13	SUBMAX	Female	47	6	1	Median duct	Yes	Yes	No
14	SUBMAX	Male	38	3	1	Median duct	Yes	Yes	No
15	SUBMAX	Female	76	4	1	Hilum	Yes	Yes	No
16	SUBMAX	Female	44	7	1	Hilum	Yes	Yes	No
17	SUBMAX	Female	44	8	1	Hilum	No	No	No
18	SUBMAX	Female	39	8	1	Hilum	No	No	No
19	SUBMAX	Male	34	5,4	2	Hilum	Yes	Yes	No
20	SUBMAX	Male	68	4	1	Hilum	Yes	Yes	No
21	SUBMAX	Male	62	3	1	Hilum	Yes	Yes	No
22	SUBMAX	Male	42	3	1	Hilum	Yes	Yes	No
23	SUBMAX	Female	36	4	1	Retrocaruncular	Yes	Yes	No
24	SUBMAX	Female	20	5	1	Hilum	Yes	Yes	No
25	SUBMAX	Male	52	7,6,4	3	Hilum	No	No	No
26	SUBMAX	Female	47	8	1	Hilum	Yes	Yes	Yes
27	SUBMAX	Male	27	8	1	Hilum	Yes	Yes	No
28	SUBMAX	Male	12	3	1	Hilum	Yes	Yes	No
29	SUBMAX	Female	32	7	1	Hilum	Yes	Yes	No
30	SUBMAX	Male	63	8	1	Hilum	Yes	Yes	No
31	SUBMAX	Female	28	6	1	Hilum	Yes	Yes	No
32	SUBMAX	Female	52	7	1	Hilum	Yes	Yes	No
33	SUBMAX	Female	61	5	1	Hilum	Yes	Yes	No
34	SUBMAX	Male	53	5	1	Hilum	Yes	Yes	No
35	SUBMAX	Male	46	4	1	Median duct	Yes	Yes	No
36	SUBMAX	Male	56	4	1	Retrocaruncular	Yes	Yes	No
37	SUBMAX	Male	60	6	1	Hilum	Yes	No	Yes
38	SUBMAX	Female	35	6	1	Hilum	Yes	Yes	No
39	SUBMAX	Female	25	6	1	Hilum	Yes	Yes	No
40	SUBMAX	Male	12	7	1	Hilum	Yes	Yes	No
41	SUBMAX	Male	12	8	1	Hilum	Yes	Yes	No
42	SUBMAX	Male	62	7	1	Hilum	Yes	Yes	No
43	SUBMAX	Female	45	6	1	Hilum	Yes	Yes	No
44	SUBMAX	Male	33	8	1	Hilum	Yes	Yes	No
45	SUBMAX	Female	40	5	1	Retrocaruncular	Yes	Yes	No
46	SUBMAX	Female	78	6	1	Hilum	Yes	Yes	No
47	SUBMAX	Female	66	7	1	Hilum	Yes	Yes	No
48	SUBMAX	Female	28	8	1	Hilum	Yes	Yes	No
49	SUBMAX	Female	52	6	1	Hilum	Yes	Yes	No
50	SUBMAX	Male	61	8	1	Median duct	Yes	Yes	No
51	SUBMAX	Male	53	7	1	Hilum	Yes	Yes	Yes
52	SUBMAX	Male	46	5	1	Hilum	Yes	Yes	No
53	SUBMAX	Male	56	6	1	Hilum	Yes	Yes	No
54	SUBMAX	Female	60	5	1	Hilum	Yes	Yes	No
55	SUBMAX	Male	35	7	1	Hilum	Yes	Yes	No
56	SUBMAX	Male	25	8	1	Median duct	No	No	No
57	SUBMAX	Male	23	7 y 6	2	Hilum	Yes	Yes	No
58	SUBMAX	Male	28	6	1	Hilum	Yes	Yes	No
59	SUBMAX	Female	45	6	1	Hilum	Yes	Yes	No
60	SUBMAX	Male	67	5	1	Median duct	Yes	Yes	No
61	SUBMAX	Male	43	5	1	Hilum	Yes	Yes	No
62	SUBMAX	Female	56	6	1	hilum	Yes	Yes	No
63	SUBMAX	Female	47	7	1	Median duct	Yes	No	Yes
64	SUBMAX	Male	23	5	1	Hilum	Yes	Yes	No
65	SUBMAX	Female	39	4,5	1	Hilum	Yes	Yes	No
66	SUBMAX	Male	21	7	1	Median duct	Yes	Yes	No
67	SUBMAX	Female	31	7	1	Hilum	Yes	Yes	No
68	PAROT	Male	19	4,5	1	Median duct	Yes	Yes	No
69	PAROT	Female	67	5	1	Median duct	Yes	Yes	Yes
70	PAROT	Female	64	6,5	1	hilum	No	No	No
71	PAROT	Female	20	3,5	1	Hilum	Yes	Yes	No
72	PAROT	Male	52	6	1	Hilum	Yes	Yes	No
73	PAROT	Female	47	5	1	Median duct	Yes	Yes	No
74	PAROT	Female	34	6	1	Hilum	Yes	Yes	No
75	PAROT	Female	19	4	1	Hilum	Yes	Yes	No
76	PAROT	Female	52	7	1	Median duct	No	Yes	No
77	PAROT	Female	63	8	1	Hilum	No	No	No
